# Synthesis, radiosynthesis and *in vitro* evaluation of ^18^F-Bodipy-C_16_/triglyceride as a dual modal imaging agent for brown adipose tissue

**DOI:** 10.1371/journal.pone.0182297

**Published:** 2017-08-17

**Authors:** Andreas Paulus, Marco Maenen, Natascha Drude, Emmani B. M. Nascimento, Wouter D. van Marken Lichtenbelt, Felix M. Mottaghy, Matthias Bauwens

**Affiliations:** 1 Department of Radiology and Nuclear Medicine, NUTRIM School for Nutrition and Translational Research in Metabolism, Maastricht University, Maastricht, The Netherlands; 2 Department of Medical Imaging, Division of Nuclear Medicine, MUMC, Maastricht, The Netherlands; 3 Division of Nuclear Medicine, Uniklinikum Aachen, Aachen, Germany; 4 Department of Human Biology & Human Movement Sciences, NUTRIM School for Nutrition and Translational Research in Metabolism, Maastricht University Medical Center, Maastricht MD, The Netherlands; Los Alamos National Laboratory, UNITED STATES

## Abstract

**Background:**

Brown adipose tissue research is in the focus in the field of endocrinology. We designed a dual-modal fluorescent/PET fatty acid based tracer on commercially available Bodipy-C_16_, which can be synthesized to its corresponding triglyceride and which combines the benefits of fluorescent and PET imaging.

**Methods:**

Bodipy-C_16_ was coupled to 1,3-diolein resulting in Bodipy-triglyceride. Bodipy-C_16_ and Bodipy-triglyceride compounds were radiolabeled with ^18^F using an ^18^F/^19^F exchange reaction to yield a dual-modal imaging molecule. Uptake of radiolabeled and non-labeled Bodipy-C_16_ and Bodipy-triglyceride was analyzed by fluorescence imaging and radioactive uptake in cultured adipocytes derived from human brown adipose tissue and white adipose tissue.

**Results:**

Bodipy-C_16_ and Bodipy-triglyceride were successfully radiolabeled and Bodipy-C_16_ showed high shelf life and blood plasma stability (99% from 0–4 h). The uptake of Bodipy-C_16_ increased over time in cultured adipocytes, which was further enhanced after beta-adrenergic stimulation with norepinephrine. The uptake of Bodipy-C_16_ was inhibited by oleic acid and CD36 inhibitor sulfosuccinimidyl-oleate. The poor solubility of Bodipy-triglyceride did not allow stability or *in vitro* experiments.

**Conclusion:**

The new developed dual modal fatty acid based tracers Bodipy-C_16_ and Bodipy-triglyceride showed promising results to stimulate further *in vivo* evaluation and will help to understand brown adipose tissues role in whole body energy expenditure.

## Introduction

Positron emission tomography (PET) allows non-invasive whole body imaging for different purposes by detecting pairs of annihilation rays. The positron emitter ^18^F is frequently used due to its short half-life (109 min), which makes it suitable for imaging purposes, and its broad domain in chemical reactions. Nevertheless PET is limited, already by physical laws (pathway of the positron), in spatial resolution and therefore cannot detect microscopic or subcellular structures. On the other hand optical fluorescence imaging has a high spatial resolution, making it an interesting topic for intraoperative imaging as well as *in vitro* evaluation of tracers [1, 2]. However, fluorescence imaging is lacking of high penetration depths. By combining PET and fluorescence imaging it is possible to overcome the disadvantages of both techniques and to create a new powerful tool to image from the whole-body down to sub-cellular level with the same imaging agent. The increased complexity and the effect of the fluorescent dye on the biodistribution are the major challenges when it comes to the development of a dual-modal imaging agent.

Brown adipose tissue (BAT) research has evolved vastly within endocrine research. For a long time it was thought that BAT was only present in infants but retrospective PET/CT studies with 2-deoxy-2-fluoro-D-glucose (FDG) identified active BAT in adult humans [3–5]. These findings could be confirmed later by dedicated cold exposure studies where a direct correlation between cold exposure and BAT metabolic activity, measured through FDG uptake, was reported [6–8]. The potential of BAT to combat obesity and obesity-associated diseases makes BAT an interesting target [9].

The variety of quantification approaches of BAT volume and metabolic activity reaches from *in vitro* experiments [10] over invasive imaging with fluorescence probes [11] or tritiated compounds [12] to non-invasive experiments with PET [3–5, 13, 14], SPECT [15, 16] and MRI [17–19]. Even though fatty acids (FAs) are the main fuel source for adipocytes, ^18^F-FDG is mostly used in studies to quantify BAT activity [4, 20, 21]. FA uptake is more difficult to quantify because there is a large variety of different FAs and triglycerides (TGs) present in the human body which makes the uptake dependent on the affinity of the single FA and not on the substance class itself. Nevertheless FAs are the major metabolized substances in BAT and therefore it is possible that BAT activity and lipid uptake is largely underestimated by FDG scans (which only show glucose-related uptake) [13]. Therefore the need exists to use a FA-based BAT tracer to quantify BAT activity and FA uptake, to study uptake dynamics and to exclude the chance of underestimating BATs metabolic activity with FDG scans.

Radiolabeled FAs in general have been developed in several variations for imaging purposes (e.g. FTHA (14(R,S)-[^18^F]fluoro-6-thia-heptadecanoic acid), BMIPP (beta-methyl-p-iodophenylpentadecanoic acid), ^11^C-palmitate [22–26]). We here report the development of a FA-based tracer which is suitable for both, PET and fluorescence imaging from the fluorescent FA Bodipy-C_16_ (BDP-FA) with which it is possible to image from whole body to sub-cellular level. Bodipy dyes have been already used to image brown adipose tissue [11] and it has been proven that fatty acid transport proteins (FATP) have a preference for Bodipy-FL coupled to a long carbon chain (C ≥ 8) [27]. Furthermore, downstream metabolic reactions in white adipocytes were already visualized [28]. Since we did not want to decrease the good binding properties of BDP-FA by introduction of another chelator molecule, we got interested in ^18^F/^19^F exchange reactions used to transform fluorescent dyes into dual-modality PET/fluorescent imaging dyes [29–33]. Because the FA is only modified at the end of the carbon chain, neither an increased steric demand, nor lowering of the targeting efficiency is expected. In comparison to previous reports, we want to go a step further and synthesize also the, *in vivo* predominant, triglyceride form of the ^18^F-BDP-FA. Here we describe a synthetic approach resulting in a dual-modal molecule to visualize BAT *in vivo* and *in vitro* with the same tracer which should help to understand this not completely evaluated tissue, its functions and metabolism.

## Materials and methods

Commercially available compounds were used without further purification unless otherwise stated. BODIPY-FL-C_16_ was purchased from Thermo Fischer Scientific (99%) (Netherlands). 1,3-diolein was purchased from Sigma Aldrich (≥ 99%). DMEM/F-12 was purchased from ThermoFischer (Waltham, MA).

All HPLC purifications (1.0 mL/min, solvent A; 0.1% TFA in water, solvent B; CH_3_CN, 50°C) were performed on a Shimadzu UFLC HPLC system equipped with a DGU-20A_5_ degasser, a SPD-M20A UV detector, a LC-20AT pump system, a CBM-20A communication BUS module, a CTO-20AC column oven, and a Scan-RAM radio-TLC/HPLC-detector from LabLogic using an Aeris™Widepore column (XB-C18, 3.6 μm, 4.6 mm × 250 mm) for the BDP-FA or an Aeris™Widepore column (C4, 3.6 μm, 4.6 mm × 250 mm) for the Bodipy-triglyceride (BDP-TG). ESI-MS was performed on a Applied Biosystems SCIEX API 150 EX electrospray ionization quadrupole (ESI-Q) mass spectrometer with the method of McAnoy et al. [34]. Briefly, 0.1M aqueous ammonium acetate solution was added to the probe to observe the ammonium salt in the MS.

^1^H-NMR spectra were carried out on a Bruker Ultrashield*TH 400 plus* at 400 MHz. Tol-d_8_ was used as solvent with TMS as internal standard. Chemical shifts are reported in parts per million (ppm) relative to the internal standard.

### Synthesis of BDP-TG 2

BDP-FA **1** (300 μg, 0.6 μmol) in acetonitrile was evaporated to complete dryness before the reactant was reconstituted in toluene (100 μL). To the resulting solution SOCl_2_ in toluene (100 μL, 4 vol.-%) was added, incubated for 5 min at 70°C in a closed vial and evaporated. The product was reconstituted in toluene (50 μL) containing 1,3-diolein (2 μL, 2.8 μmol) and heated to 100°C for 30 min. After the reaction time, purification by HPLC (1 mL/min, 30% to 15% A in 5 min, 15% to 0% A from 5 to 6 min, 0% A to 20 min) yielded **2** (225 μg, 75%) as a red solid; t_R_ = 12.3 min. ESI-MS(+): m/z (%) = 1058 (100) [M—F^-^]^+^, 1095 (82) [M + NH_4_]^+^. ^1^H NMR (400 MHz, Tol-d_8_); δ (ppm) = 5.46 (m, 4H), 4.26 (m, 2H), 4.06 (m, 2H), 3.13 (m, 1H), 1.75 (s, 3H).

Additional experiments were performed using BMIPP (beta-methyl-p-iodophenylpentadecanoic acid) as starting FA. Different ways of synthesis were evaluated, where reaction time, temperature and chlorinating agent were changed (see results section).

### Radiolabeling of Bodipy-C_16_ 3

Aqueous ^18^F solution was loaded on a QMA-cartridge which was preconditioned with 15 mL K_2_CO_3_ in water and 20 mL water. Fluoride (42 MBq) was eluted with a mixture of 600 μL acetonitrile, 300 μL H_2_O and 100 μL K_2_CO_3_ solution (5 mg/mL). ^18^F solution was transferred into a drying vessel containing tetra-n-butylammonium bromide (80 μL) as a phase transfer agent. Acetonitrile (3 × 1.0 mL) was added and the solution of ^18^F was dried by heating to 100°C with a continuous flow of argon. After reconstitution of ^18^F in anhydrous acetonitrile (100 μL), a solution of **1** (50 μg, 0.1 μmol) and SnCl_4_ (0.2 M in acetonitrile, 100 μL) was added to the activity solution and the reaction was stirred at r.t. for 30 min. After addition of water (200 μL) and filtration (Millex, hydrophile PVDF 0.22 μm) a quality control was performed by HPLC (1 mL/min, 30% to 15% A in 5 min, 15% to 0% A from 5 to 6 min, 0% A to 20 min) and afforded **3** (decay corrected radiochemical yield (RCY): 76%, 22 MBq) with a decay corrected specific activity of 220 MBq/μmol and a radiochemical purity of ≥ 99%; t_R_ = 13.3 min. In addition a TLC with Toluene, CHCl_3_ and MeOH (80.9%, 14.3%, 4.8%) was performed.

### Radiolabeling of Bodipy-TG 4

Drying process was performed as mentioned in previous section. After reconstitution of ^18^F (83 MBq) in anhydrous acetonitrile (100 μL), a solution of **2** in toluene (107 μg, 0.1 μmol in 50 μL) and SnCl_4_ (0.2 M in acetonitrile, 100 μL) was added to the solution with the activity and the reaction solution was stirred at room temperature (r.t.) for 30 min. After addition of water (200 μL), filtration and washing with water (2 x 200 μL) a quality control was performed by HPLC (1 mL/min, 30% to 15% A in 5 min, 15% to 0% A from 5 to 6 min, 0% A to 20 min) and afforded **4** (decay corrected RCY: 44%, 25 MBq) with a decay corrected specific activity of 250 MBq/μmol and a radiochemical purity of > 95%; t_R_ = 12.5 min. In addition TLC with toluene, CHCl_3_ and MeOH (80.9%, 14.3%, 4.8%) was performed.

### Human primary adipocyte cultures derived from BAT and white adipocytes (WAT)

The isolation and differentiation of human adipocytes has been described before [35]. The study was reviewed and approved by the ethics committee of Maastricht University Medical Center (METC 10-3-012, NL31367.068.10). Informed was obtained prior to surgery. The precursor cells were obtained from a stromal vascular fraction of BAT and WAT tissue. The sample was taken from a 34 year old male with a benign formation in the left thyroid gland. The cells have been metabolically characterized [35]. In short, the stromal vascular fraction was obtained from BAT and subcutaneous WAT biopsies from the same individual undergoing deep neck surgery. Collected cells were grown to confluence prior to start of differentiation in DMEM/Ham’s F-12 (Gibco) supplemented with 10% Fetal Bovine Serum (Bodinco BV). Differentiation medium composed of biotin (33 μM), D-pantothenate (17 μM), h-insulin (100 nM), dexamethasone (100 nM), IBMX (250 μM), rosiglitazone (5 μM), T3 (2 nM), transferrin (10 μg/ml). After 7 days of differentiation, the medium was exchanged for maintenance medium composed of biotin (33 μM), pantothenate (17 μM), insulin (100 nM), dexamethasone (10 nM), T3 (2 nM), and transferrin (10 μg/ml).

### *In vitro* uptake of 3 and 4 in cultured adipocytes derived from human BAT and WAT

Adipocytes derived from human BAT and WAT were incubated with **3** and **4** (1 nM, 100 μL) for 1 h. **3** was added in 0.5% fatty acid free bovine albumin in DMEM/F-12. **4** was dissolved in 50% PEG in DMEM/F-12. Adipocytes were exposed to the tracer or preincubated with sulfosuccinimidyl oleate (500 μM, 100 μL, 30 min) or norepinephrine (1 μM, 100 μL, 30 min). Radioactivity was quantified by measuring washed, detached cells with a WIZARD^2^ automatic γ-counter from Perkin Elmer. Number of cells was determined by three extra wells which underwent a standard cell count protocol using a LUNA II (Logosbio) automated cell counter.

### *In vitro* imaging of cultured adipocytes derived from human BAT and WAT

Adipocytes derived from human BAT and WAT were incubated with **1** and **2** (0–4 μM, 500 μL) for different time points (1–24 h) in experimental medium. **1** was added in 0,5% fatty acid free bovine albumin in DMEM/F-12. **2** was dissolved in 50% PEG in DMEM/F-12. Adipocytes were exposed to the tracer or coincubated with oleic acid (400 μM, 500 μL) or preincubated with sulfosuccinimidyl-oleate (500 μM, 500 μL, 30 min). After incubation adipocytes were fixed in 3.7% formaldehyde (4°C, 30 min) and stained with DAPI (4′,6-diamidino-2-phenylindole) (RT, 5 min). The adipocytes were imaged with a Sony Eclipse e800 fluorescence camera. The signal was measured in the FITC channel (460–490 nm excitation, 510–550 nm emission) and in the DAPI channel (385–415 nm excitation, 450–470 nm emission). Quantification of signal was performed with Fiji [36] as well as correction for exposure time, background and cell auto-fluorescence. For every data point at least three cells were analyzed. Graphs and statistical analysis was performed using GraphPad Prism 6.

### Fluorescence measurements (microplate reader)

Adipocytes derived from human BAT cultured on 96 well chamber slides were incubated with **1** and **2** (2 μM, 100 μL) for different time points (1–4 h). **1** was added in 0,5% fatty acid free bovine albumin in DMEM/F-12. **2** was dissolved in 50% PEG in DMEM/F-12. Adipocytes were exposed to the tracer or preincubated with norepinephrine (1 μM, 500 μL, 30 min). After incubation adipocytes were washed 3 times (30 seconds each) with cold PBS and suspended in PBS. The signal was measured using a SpectraMax M2 plate reader (molecular devices) (excitation 485 nm, emission 520 nm). Graphs and statistical analysis was performed using GraphPad Prism 6.

### *Ex vivo* stability

Shelf life of **3** was investigated under following conditions: 24 MBq (60 μL) were added to H_2_O (1 mL) and integrity was examined by HPLC (1–4 h). Areas under the peak of free ^18^F and metabolites were compared to the area under the peak of **3**.

Plasma stability was examined by adding 28 MBq (60 μL) of **3** to 1 mL of human plasma (37°C). After certain time points (20, 40, 60 min and 2–4 h) integrity of the compound was analyzed by HPLC.

Determining the aqueous or plasma stability of the radiolabeled TG **4** failed due to the insolubility of the compound in aqueous medium.

## Results

**Fig 1:**
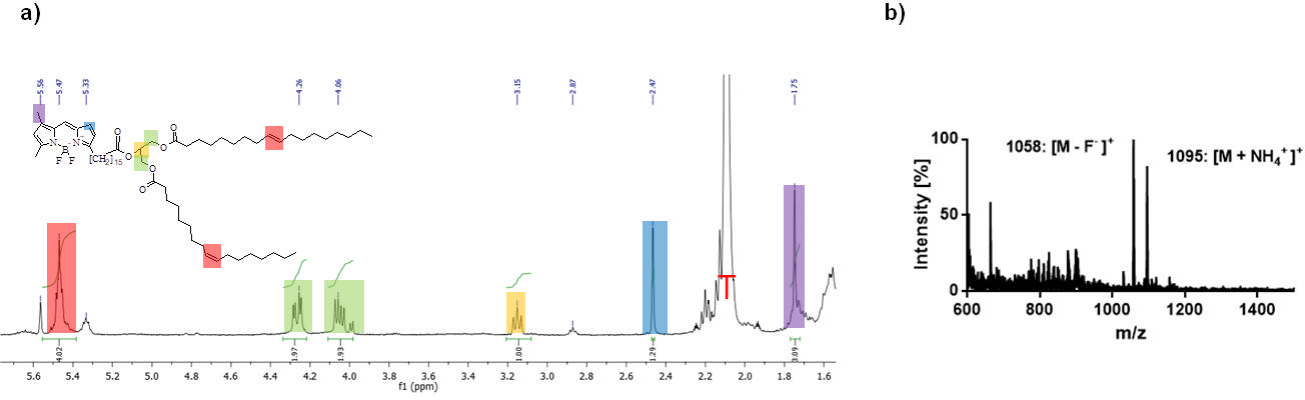
**BDP-TG analysis a** ^1^H-NMR of BD-TG in toluene-d5, red T corresponds to the toluene peek **b** ESI-MS of BDP-TG with NH_4_OAC in Chloroform/MeOH 50:50.

Different conditions for the esterification of the BDP-C_16_ to BDP-TG were tested (Table 1). The best yield, analyzed by HPLC injection of the crude reaction mixture, was achieved with BDP-C_16_ in Toluene (50 μL) and the strong chlorinating agent thionylchloride at 70°C in 30 min. More hydrophilic solvents, larger reaction volumes, lower temperatures or milder chlorination conditions resulted in lower yields and/or byproducts. In optimal conditions, after reaction and purification **2** was obtained in a yield of 75%. After purification of **2** further analysis of quenching effects was performed by HPLC. Both, FA and TG, showed similar absorption when the same molar amount was injected. NMR of **2** confirmed the identity of the TG (Fig 1A and S4 Fig). The disappearance of the alcohol function (3.75 ppm) with the simultaneous formation of a third ester bond (3.15 ppm) plus the additional BDP peaks (1.75 ppm and 2.47 ppm) proved the successful synthesis of **2**. Additionally ESI-MS of **2** supports these findings as peaks found in the spectrum correspond with the calculated molecular masses (Fig 1B).

**Table 1:** Different esterification conditions for BMIPP or BDP-C16. Yields tested by injection of the crude reaction mixture into HPLC and comparison of the esterified vs. the non-esterified FA signal plus byproducts.

Fatty acid	Coupling agent	Solvent	Rec. volume [μL]	Activator	Temperature [°C]	Reaction time [min]	Yield [%]	n
BMIPP	1,3-Diolein	Toluene	50	SOCl_2_	70	30	72	4
BMIPP	1,3-Diolein	Acetonitrile	50	SOCl_2_	70	30	0.6	1
BMIPP	1,3-Diolein	Diethylether	50	SOCl_2_	70	30	67	1
BMIPP	1,3-Diolein	Tetrachlormethane	50	SOCl_2_	70	30	8	1
BMIPP	1,3-Diolein	Benzene	50	SOCl_2_	70	30	38	1
BMIPP	1,3-Diolein	Toluene	50	Oxalyl Chloride	70	30	43	1
BMIPP	1,3-Diolein	Toluene	50	Oxalyl Chloride	70	60	44	1
BMIPP	1,3-Diolein	Toluene	50	SOCl_2_	70	90	54	2
BMIPP	1,3-Diolein	Toluene	50	SOCl_2_	70	120	67	2
BMIPP	1,3-Diolein	Toluene	50	SOCl_2_	70	150	86	2
BMIPP	EtOH	MeCN	100	SOCl_2_	0	30	1	1
BMIPP	EtOH	MeCN	100	SOCl_2_	22	30	64	1
BMIPP	EtOH	MeCN	100	SOCl_2_	70	30	88	1
Bodipy C_16_	1,3-Diolein	Toluene	200	SOCl_2_	70	30	59	1
Bodipy C_16_	1,3-Diolein	Toluene	50	SOCl_2_	70	30	95	8

**Fig 2:**
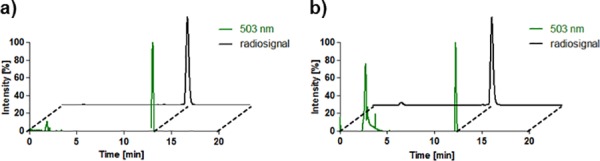
**Chromatograms for BDP-FA/TG a** HPLC chromatograms (radio trace and absorbance at 503 nm) of ^18^F-BDP-FA **3** on a C18 column. **b** HPLC chromatograms (radio trace and absorbance at 503 nm) of ^18^F-BDP-TG 4 (peak at 3 min corresponds to toluene which was used to solubilize the probe) on a C4 column.

The radiolabeled dual-modality imaging agent ^18^F-BDP-TG was synthesized in a two-step procedure. First the Bodipy-FA **1** was esterified to BDP-TG **2** and then radiolabeled with ^18^F using the strong Lewis acid SnCl_4_ in dry acetonitrile/toluene. After 30 min of incubation, ^18^F-BODIPY-TG **4** was isolated by washing 3 times with water allowing 44% radiochemical yield, a specific activity of 250 MBq/μmol and a radiochemical purity of > 95% (Fig 2B). First approaches to radiolabel the BDP-FA and perform the esterification afterwards resulted in higher labeling yields (76%) (Fig 2A) in the first step but the following esterification afforded only small amounts of radiolabeled BDP-TG. The radiolabeling was performed in acetonitrile but in previous cold esterification attempts it was shown that only small yields can be reached in this solvent (Table 1). A complete exchange of acetonitrile to toluene is time consuming as well as the problem of ^18^F-BDP-FA getting attached to the glass vial arises. Different reaction conditions for the radiolabeling can be found in Table 2. Shelf life and plasma stability showed 99% intact radiolabeled compound **3** after 4 h (S5 Fig).

**Table 2:** Radiolabeling of BDP-FA 3 and BDP-TG 4. Different conditions tested for both compounds. Radiochemical yields calculated after purification.

Agent	V (SnCl_4_) [μL]	Solvent	Volume [μL]	Reaction time [min]	RCY [%]	n
Bodipy C_16_	100 (0.1M)	MeCN + K_222_ + K_2_CO_3_	250	30	7.3	4
Bodipy C_16_	30 (0.1M)	MeCN + K_222_ + K_2_CO_3_	180	30	13.8	2
Bodipy C_16_	100 (0.2M)	MeCN + K_222_ + K_2_CO_3_	250	30	28	4
Bodipy C_16_	100 (0.3M)	MeCN + K_222_ + K_2_CO_3_	250	30	0	1
Bodipy C_16_	100 (0.2M)	MeCN + K_2_CO_3_	250	30	76	9
Bodipy-Tg	100 (0.2M)	MeCN + K_2_CO_3_/DMA	250	30	0	1
Bodipy-Tg	100 (0.2M)	MeCN + K_2_CO_3_/Toluene	250	30	44	7

**Fig 3:**
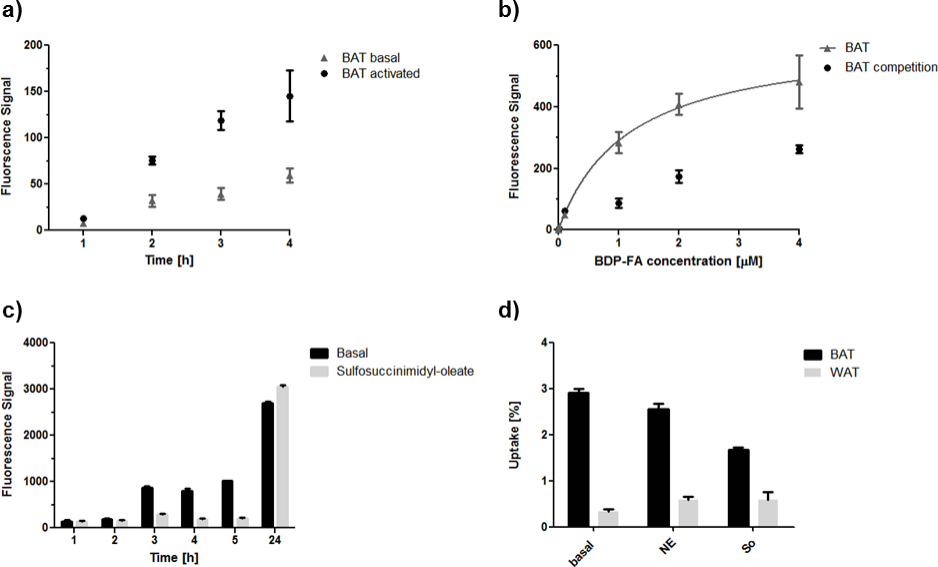
***In vitro cell* uptake a)** uptake time dependence measured with 2 μM **1** in BAT with and without activation with norepinephrine (microplate) **b)** uptake concentration dependence measured in BAT over 2 h with and without coincubation with oleic acid (fluorescence microscope) **c)** BAT blocking study over time with sulfosuccinimidyl-oleat (fluorescence microscope) **d)** uptake of **3** in BAT and WAT cells under basal conditions, activation with Norepinephrine (NE) and blocking with sulfosuccinimidyl-oleat (So) after 1 h incubation time.

Fluorescent uptake of **1** was evaluated *in vitro* with cultured adipocytes derived from human BAT. Adipocytes were incubated with BDP-FA (2 μM) for 1 to 4 h and fluorescence was measured with a microplate reader (SpectraMax M2) (Fig 3A). An increase over time was observed where BAT took up 274%, 362% and 697% more after 2 h, 3 h and 4 h compared to the 1 h time point. Activation with norepinephrine (1 μM, 30 min before incubation with BDP-FA) increased BAT uptake of **1** by 134%, 199% and 143% after 2, 3 and 4 h compared to their basal uptake values at these time points. Only after 1 h no significant increase was observed. Single cell uptake was analyzed and quantified with a fluorescence microscope (Sony Eclipse e800) under different conditions (S1 Fig). Concentration dependent uptake was examined within a 2 h time period (Fig 3B). A clear Michaelis—Menten like kinetic was observed in uptake-positive BAT cells (with k_m_ = 1.15 μM, R^2^ = 0.93). Coincubation with 400 μM oleic acid reduced uptake of BDP-FA by 46%.

Time dependence with simultaneous inhibition of the cluster of differentiation 36 (CD36), which is a scavenger protein for long chain fatty acids [37] and was shown to be essential for thermogenesis in animals during fasting conditions [16], was additionally quantified on a cellular level (Fig 3C) (S1 Fig). By preincubation with sulfosuccinimidyl-oleate a blocking of 73% ± 6% was reached after 3–5 h. After 24 h the uptake recovered to basal conditions.

No uptake was observed by incubation with **2** (2 μM, 24 h) (S3 Fig).

Cell uptake experiments were also performed in WAT cells to further analyze the suitability of the BDP-FA as a tracer for adipocytes and to compare BAT and WAT cells. The WAT cells were obtained from the same human sample as the BAT cells.

In first microplate activation experiments it was shown that, similar to BAT, the uptake of **1** increased over time. After 2 h, 3 h and 4 h the uptake increased by 221%, 294% and 357%. Activation of the cells by norepinephrine resulted in an increase in uptake between 10% and 48% and was significant lower compared to BAT cells.

In WAT cells incubated with **1** (0–4 μM) for different concentrations, a Michaelis—Menten like kinetic was observed in uptake-positive cells (with k_m_ = 1.58 μM and R^2^ = 0.97). The uptake was also reduced with an excess of oleic acid (400 μM) by 53%.

CD36 dependency on uptake kinetics was also investigated in WAT cells (S2 Fig). Sulfosuccinimidyl-oleat only had a significant negative effect on the uptake of the 2 h and 3 h group (51% ± 16%) but in general the uptake kinetics of the preincubated cells with sulfosuccinimidyl-oleat followed more the kinetics of the cells under basal conditions.

BAT and WAT uptake of **3** and **4** was investigated to prove the functionality of the radiolabeled compounds and to quantify the total uptake of both tracers. Interestingly, in this experimental setup, BAT took up in all cases more FAs than WAT (Fig 3D). After 1 h WAT took up 0.3% ± 0.05% and BAT 2.9% ± 0.09%. Activation of WAT with norepinephrine showed a small increase in uptake (0.6% ± 0.07% vs. 0.3% ± 0.05%) whereas the uptake in BAT was slightly decreased (2.6% ± 0.11% vs. 2.9% ± 0.09%). An effect could be observed for preincubation with sulfosuccinimidyl-oleat in BAT after 1 h (1.7% ± 0.04% vs. 2.9% ± 0.09%). WAT showed no significant decrease (0.58% ± 0.18% vs. 0.3% ± 0.05%).

Incubation experiments with the radiolabeled TG **2** were conducted additionally but showed no consistent and reproducible results (data not shown).

## Discussion

Imaging of BAT has evolved to an interesting and fast developing topic in endocrine research. Several imaging approaches have been used in the past to visualize and quantify BAT and its metabolic activity reaching from *in vitro* experiments [10] over invasive imaging with fluorescence probes [11] or tritiated compounds [12] to non-invasive experiments with PET [3–5, 13, 14], SPECT [15, 16] and MRI [17–19]. Most often ^18^F-FDG scans are used for BAT imaging. This has the big disadvantage that it only shows glucose-related uptake and has the chance to underestimate BAT activity because the major metabolized substance class in BAT is FAs. There is therefore a need for a FA BAT tracer to quantify metabolic activity in a more precise way. In studies with FTHA it was observed that radiolabeled FAs showed an increased uptake in BAT under cold stimulation in humans [13]. With our developed tracer it is possible to examine uptake characteristics with both PET and fluorescence imaging. Therefore scans from subcellular level, to determine the localization of the probe within the cell, to whole body scans are possible.

*In* vivo FAs are transported as TGs in lipoproteins towards adipocytes [38, 39]. We were able to produce BDP-TG in a very reasonable yield (77%). Both, the FA and the TG analog, were successfully radiolabeled (RCY: 76% for ^18^F-BDP-FA and 44% for ^18^F-BDP-TG), comparable to previously achieved yields with other modified Bodipy-dyes by other groups [31, 40]. Activation of ^18^F by K_2_CO_3_ and K_222_ (4,7,13,16,21,24-Hexaoxa-1,10-diazabicyclo[8.8.8]hexacosane) resulted in side products and decomposition of the Bodipy-dye. Reactions without addition of a base, such as K_2_CO_3_, gave lower labeling yield. Shelf life and plasma stability of ^18^F-BDP-FA indicated its suitability as an *in vivo* imaging agent, although these values could not be determined for ^18^F-BDP-TG due to its insolubility in aqueous medium. Nevertheless no large stability deviations are expected for ^18^F-BDP-FA and ^18^F-BDP-TG, as the lipid backbone should not affect the boron-fluoride bond in a large extend. Next to our synthesis work we put effort in first *in vitro* application of our cold tracer. Uptake experiments proved that the BDP-FA is taken up with the characteristics of a regular FA (k_m_ = 1.15 μM compared to k_m_ = 0.2 μM of oleate [41] and cis-parinaric acid: k_m_ = 1.5 μM in 3T3 fibroblasts [42]) and uptake could be decreased by coincubation with oleic acid in excess. BAT cells were sensitive to norepinephrine and uptake was increased by a preincubation with this hormone. Sulfosuccinimidyl-oleat as an antagonist blocked CD36 and overall uptake of BDP-FA was decreased to a minimum. This shows that CD36 is essential for the lipid uptake in BAT cells and is in accordance to already published *in vivo* results [43, 44]. Only after 24 h the cells were able to overcome this blocking procedure and showed comparable uptake to basal conditions. Stahl et al. found over 90% of CD36 in serum starved adipocytes on the cell membrane [42]. Therefore it can be speculated that the increase in uptake resulted in a displacement of sulfosuccinimidyl-oleat at this time point.

The uptake in WAT cells of BDP-FA was also analyzed. Comparable to BAT cells fluorescence signal increased over time and uptake was partially blocked by coincubation with an excess of oleic acid. Neither the positive uptake effect in cells preincubated with norepinephrine nor the negative uptake effect in cells preincubated with sulfosuccinimidyl-oleat was observed in such an extent in WAT compared to BAT. Therefore a smaller amount of β3-adrenoreceptors on the cell surface [45] and a CD36 independent uptake mechanism [46] is proposed for WAT cells.

In former experiments (data not shown) it was observed that the FA and the TG signal are significantly quenched in an aqueous medium. This is in accordance with other published results for the BDP-FA [47]. A self-quenching effect for different Bodipy-dyes depending on their solvent solubility has also been reported before [48]. This makes fluorescent imaging with this tracer susceptible for misinterpretation because one can underestimate the total uptake. Nevertheless we could easily and without any dose characterize, but not quantify, *in vitro* uptake kinetics of this fluorescent FA.

Even though first indications on uptake mechanism in brown and white adipocytes were obtained by fluorescent experiments, radiolabeled compounds were necessary to quantify cell uptake and to exclude the chance of a quenched fluorescent signal. Cell uptake experiments with the radiolabeled FA showed that after correction for different cell numbers, BAT took up significantly more ^18^F-BDP-FA than WAT. Sulfosuccinimidyl-oleat decreased the uptake significantly after 1 h in the BAT group. By preincubation with norepinephrine the uptake in BAT was not affected (as seen in the fluorescent experiments, increased uptake could only be observed 2 h after stimulation). In WAT no significant effect was observed for the preincubation with sulfosuccinimidyl-oleat. Preincubation with norepinphrine resulted in the same uptake value as preincubation with sulfosuccinimidyl-oleat, which denotes to a norepinephrine insensitive and CD36 independent uptake mechanism. Overall, the results obtained with the radiolabeled BDP-FA are in accordance with the results obtained in the fluorescent experiments. By this method it was possible to quantify uptake values in percent uptake and a comparison between both cell types could be performed.

For the TG no uptake was observed with the presented methods. This might be due to the insolubility of TG in aqueous solutions and it is therefore not available for uptake by adipocytes. Alternatively, another reason could be that lipoprotein lipase, which is responsible for TG hydrolysis, is only activated by lipoproteins, which was not present in our in vitro experiments.

A possible solution for the inaccessibility of the BDP-TG tracer, non-radiolabeled and radiolabeled, for our *in vitro* cell model would be the incorporation of this tracer into lipoproteins. As TG are transported *in vivo* in these water soluble particles an incorporation of our tracer would 1) simulate the physiologic state how TG are transported and 2) overcome the limited solubility of the compound, which also limits the *in vivo* application. With other FA-tracers (mainly tritiated) this has already been achieved and *in vivo* experiments have been performed [11, 12]. In comparison to tritiated compounds, the here presented tracer has the advantage that it can be visualized and quantified by PET *in vivo* and its uptake mechanism and kinetics can be followed by fluorescence microscopy *in vitro*.

To conclude, we present the successful development of a multimodal FA/TG BAT tracer. We were able to conjugate BDP-C16 to 1,3-diolein and radiolabel this triglyceride. We then investigated its uptake characteristics *in vitro* with fluorescence imaging in a human BAT and WAT sample, and observed CD36 mediated uptake which was sensitive to norepinephrine in BAT cells. Additionally, we showed that BAT takes up significantly more FAs than WAT in our *in vitro* radioactive uptake experiments.

Future *in vitro/vivo* experiments are required with ^18^F-Bodipy-TG, incorporated into a chylomicron, where these first insights in brown adipose tissue metabolism will help to specify the role of this interesting tissue for whole body energy metabolism.

## Supporting information

S1 Fig Uptake of 1 in BAT images obtained from fluorescence microscope by incubation with BDP-FA under basal conditions, and sulfosuccinimidyl–oleat incubation. Green (Bodipy-signal): 460–490 excitation, 510–550 emission, Blue (Dapi-signal): 385–415 excitation, 450–470 emmision.
(TIF)

S2 Fig Uptake of 1 in WAT images obtained from fluorescence microscope by incubation with BDP-FA under basal conditions and sulfosuccinimidyl–oleat incubation. Green (Bodipy-signal): 460–490 excitation, 510–550 emission, Blue (Dapi-signal): 385–415 excitation, 450–470 emmision.
(TIF)

S3 Fig Uptake of 2 in WAT and BAT images obtained from fluorescence microscope by incubation with BDP-TG (2 μM, 24 h) under basal conditions. Green (Bodipy-signal): 460–490 excitation, 510–550 emission, Blue (Dapi-signal): 385–415 excitation, 450–470 emmision.
(TIF)

S4 Fig NMR analysis. NMR spectra of BDP-TG **1**(a) and 1,3-Diolein (b), alcohol function of the Diolein is shown in red square, formed triple ester bond is shown in blue square. No significant impurities were noted.
(TIF)

S5 Fig Shelf life and plasma stability of 3. (TIF)
