# Supplementary figures and images for: Synthesis, radiosynthesis and in vitro evaluation of 18F-Bodipy-C16/triglyceride as a dual modal imaging agent for brown adipose tissue
Source: PLoS One. 2017 Aug 17;12(8):e0182297. doi: 10.1371/journal.pone.0182297 (PMC5560730; doi:10.1371/journal.pone.0182297)

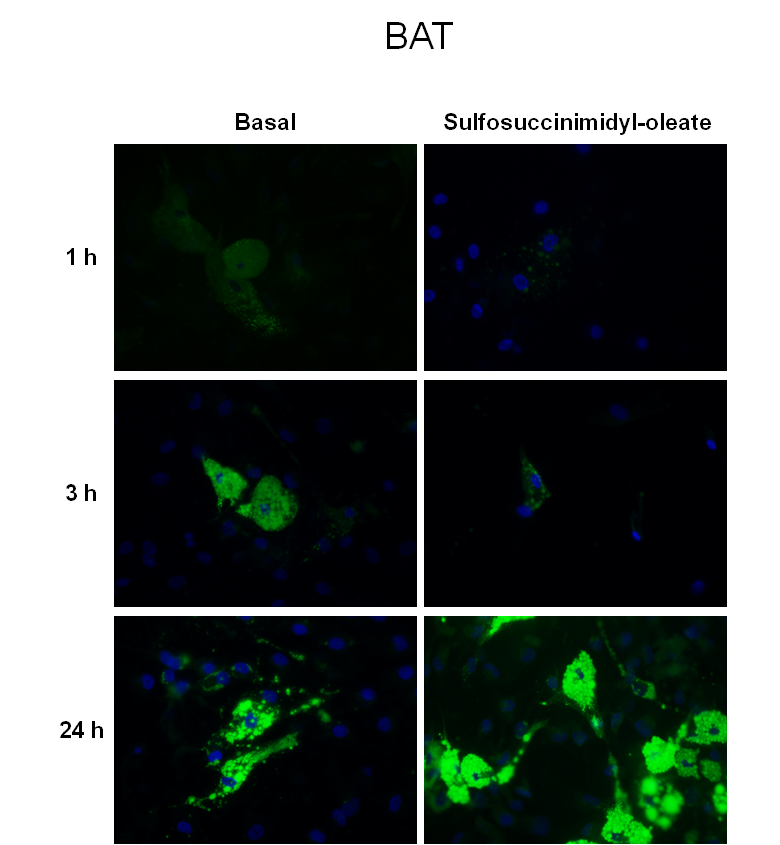

Supplement: S1 Fig — Green (Bodipy-signal): 460–490 excitation, 510–550 emission, Blue (Dapi-signal): 385–415 excitation, 450–470 emmision. (TIF) [file pone.0182297.s001.tif]

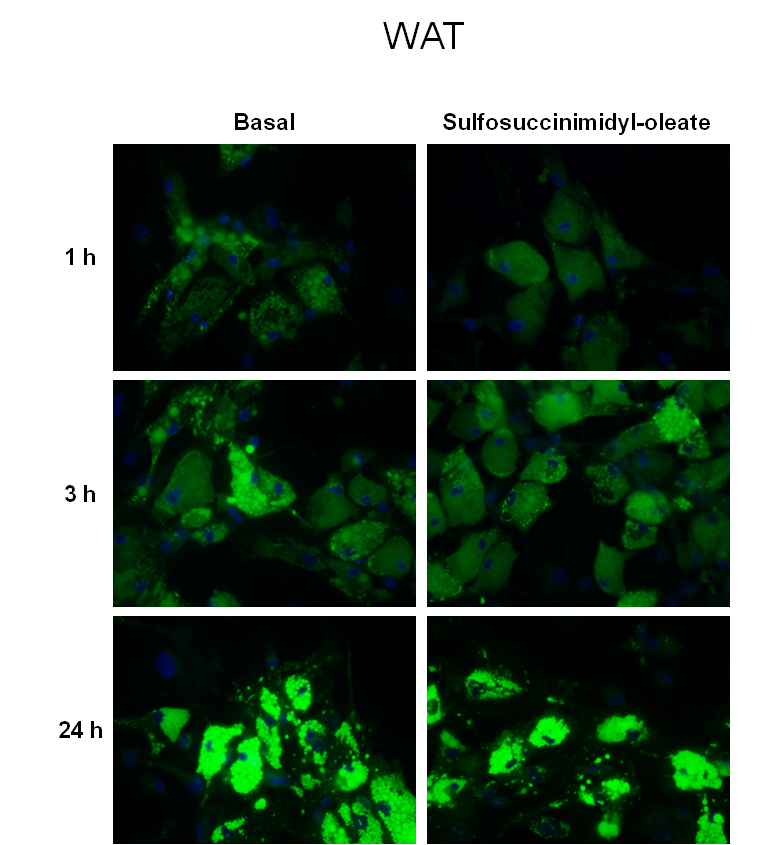

Supplement: S2 Fig — Green (Bodipy-signal): 460–490 excitation, 510–550 emission, Blue (Dapi-signal): 385–415 excitation, 450–470 emmision. (TIF) [file pone.0182297.s002.tif]

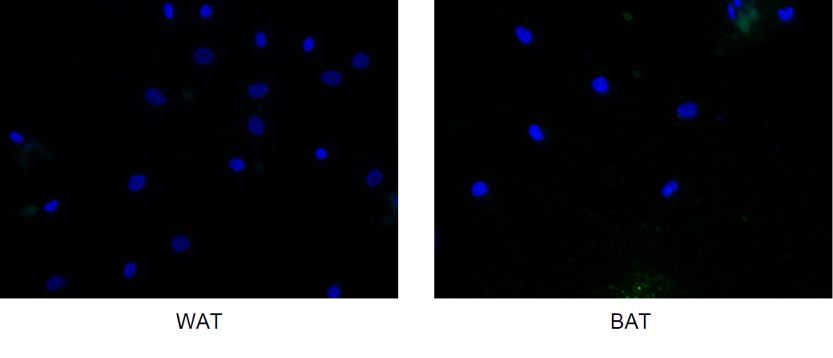

Supplement: S3 Fig — Green (Bodipy-signal): 460–490 excitation, 510–550 emission, Blue (Dapi-signal): 385–415 excitation, 450–470 emmision. (TIF) [file pone.0182297.s003.tif]

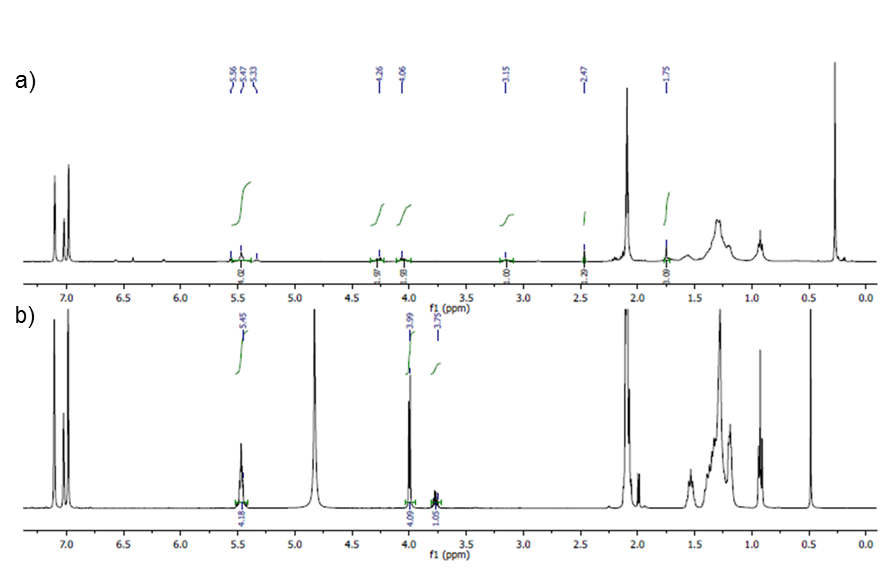

Supplement: S4 Fig — NMR spectra of BDP-TG 1(a) and 1,3-Diolein (b), alcohol function of the Diolein is shown in red square, formed triple ester bond is shown in blue square. No significant impurities were noted. (TIF) [file pone.0182297.s004.tif]

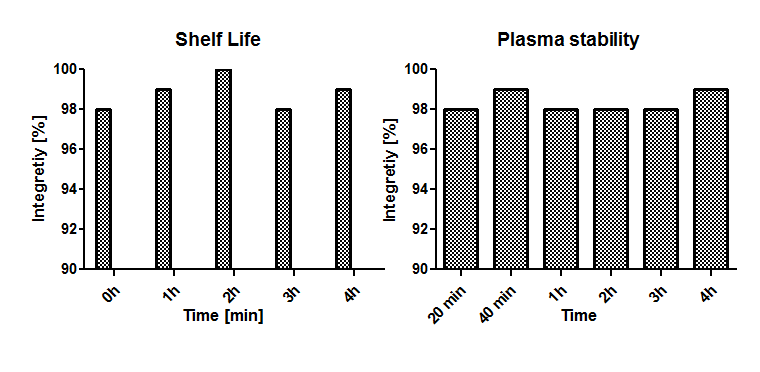

Supplement: S5 Fig — (TIF) [file pone.0182297.s005.tif]
